# Adherence to secondary preventive treatment following myocardial infarction with and without obstructive coronary artery disease

**DOI:** 10.1371/journal.pone.0324072

**Published:** 2025-05-23

**Authors:** Anna M. Nordenskjöld, Miriam Qvarnström, Björn Wettermark, Bertil Lindahl

**Affiliations:** 1 Department of Cardiology, Faculty of Medicine and Health, Örebro University, Örebro, Sweden; 2 Department of Pharmacy, Faculty of Pharmacy, Uppsala University, Uppsala, Sweden; 3 Department of Medical Sciences, Uppsala University, Uppsala, Sweden; Tan Tao University, VIET NAM

## Abstract

**Background:**

Secondary preventive medications following myocardial infarction (MI) reduce the risk of new cardiovascular events. Discontinuation and suboptimal adherence are common and affect prognosis. However, there is limited knowledge regarding adherence in patients with myocardial infarction with non-obstructive coronary arteries (MINOCA). We therefore aim to evaluate the adherence to guideline recommended medications in patients with MINOCA and myocardial infarction with obstructive coronary arteries (MI-CAD).

**Methods:**

This was a Swedish nationwide observational study of MI patients recorded in the SWEDEHEART registry between 2006─2017. A total of 9,138 MINOCA and 107,240 MI-CAD patients were followed for a mean 5.9 years. Initiation of therapy, implementation determined using medication possession ratio, and persistence rates during different time periods were calculated.

**Results:**

Patients with MINOCA were less frequently prescribed secondary preventive medications than MI-CAD. The percentage of patients taking medication as prescribed were lower in MINOCA than in MI-CAD at all time points; during months 6─12 after discharge: aspirin 94.8% vs 97.2% (p < 0.001), statins 90.3% vs 94.7% (p < 0.001), and ACEI/ARBs 97.7% vs 98.5% (p = 0.002) and at 12 months: aspirin 84.4% vs 93.7% (p < 0.001), statins 83.8% vs 94.8% (p < 0.001), ACEI/ARBs 85.0% vs 92.2% (p < 0.001) and beta blockers 80.4% vs 89.6% (p < 0.001).

**Conclusion:**

The rates of initiation, implementation, and persistence of secondary preventive medications were high in both MINOCA and MI-CAD patients during the first 5 years after MI. The lower rates in patients with MINOCA may be partially due to uncertainties regarding the diagnosis of MINOCA, differences in patient characteristics, and psychosocial factors. Suboptimal medical adherence in patients with MINOCA may adversely affect prognosis as previously demonstrated in MI-CAD patients.

## Introduction

Outcomes after acute myocardial infarction (MI) can be improved by lifestyle changes; control of cardiovascular risk factors; and treatment with secondary preventive medications, such as aspirin, P2Y12-inhibitors, statins, beta blockers, angiotensin-converting enzyme inhibitors (ACEIs), and/or angiotensin-receptor blockers (ARBs), all of which are recommended in international guidelines [1–3].

Suboptimal treatment after MI has been repeatedly observed, with too few patients initiated on recommended secondary preventive treatments and many patients showing insufficient adherence to medication [4–13]. Poor adherence to prescribed secondary preventive drugs has been found to adversely affect prognosis [6,9–12].

About 6–8% of patients who experience MI are diagnosed with myocardial infarction with non-obstructive coronary arteries (MINOCA) [14,15]. Although this disorder was first recognized in the early 1980’s [16–18], diagnostic criteria and treatment recommendations for MINOCA have only recently been established [2,19,20]. An AHA scientific statement from 2019 suggests that secondary preventive therapies might be considered on an individual basis in patients with MINOCA [20]. The guidelines from European Society of cardiology from 2020 recommend that patients with MINOCA, of unknown cause, might be followed-up similarly to patients diagnosed with MI with obstructive coronary arteries (MI-CAD), and be treated according to secondary prevention guidelines for atherosclerotic disease (class IIb recommendation) [2]. Recommendation on duration of the treatment is however scarce. The percentage prescribed secondary preventive drugs has been shown to be lower in patients with MINOCA than in those with MI-CAD in clinical routine [21,22]. However, knowledge is lacking regarding adherence to medical treatment in patients with MINOCA and whether the different medication adherence measures, including initiation, implementation, and persistence rates of secondary preventive drug treatment differ between patients with MINOCA and MI-CAD. We therefore aim to evaluate the adherence to guideline recommended medications in patients with MINOCA and MI-CAD.

## Methods

### Patient selection

**Fig 1:**
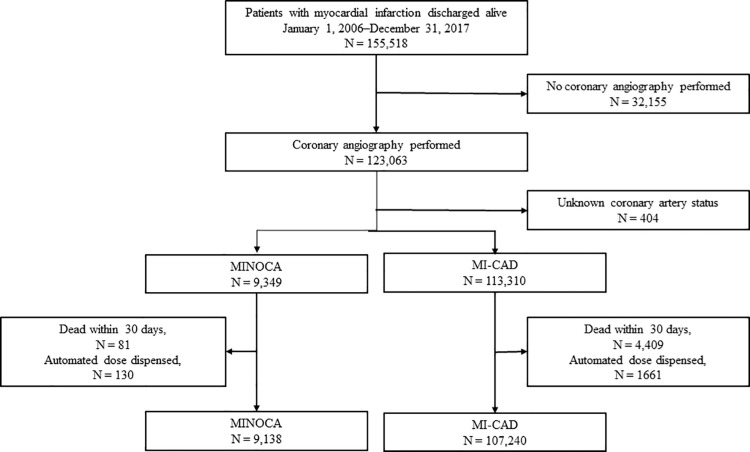
Study population. Patients were excluded if they did not undergo in-hospital diagnostic coronary angiography, if their result of the coronary angiography is unknown, died within 30 days after discharge, or were automatically dispensed doses of medication. Patients with previous PCI or CABG were considered to have a MI-CAD.

The present study is a Swedish nationwide register-based cohort study, based on the 155,518 unique patients in the SWEDEHEART registry [23], who were hospitalised due to acute MI and discharged between January 1, 2006 and December 31, 2017. Patient with at least one coronary stenosis ≥50% at coronary angiography were labelled MI-CAD and patients without were labelled MINOCA. Patients were excluded if they did not undergo in-hospital diagnostic coronary angiography, if their result of the coronary angiography was unknown, died within 30 days after discharge, or were receiving automatically dispensed doses of medication before admission to hospital. Patients who previously underwent percutaneous coronary intervention (PCI) or coronary artery bypass grafting (CABG) were included in the MI-CAD group independently on findings at the latest coronary angiography. The final study cohort consisted of 116,378 individuals, 9138 with MINOCA and 107,240 with MI-CAD (Fig 1). Patient were followed from the date of hospital discharge to the date of death or end of the study period, whichever occurred first. Patients were censored at death or and at the end of the study period.

### Data sources

This study used data from three Swedish national registries linked through the unique social security number that all Swedish citizens have. The data from SWEDEHEART were merged with census data (migration and death) for the Swedish population and two Swedish population-based mandatory national registries maintained by the National Board of Health and Welfare: the ‘Patient Register,’ which includes all ICD-codes for all hospital admissions [24], and the ‘Prescribed Drug Register,’ which contains data from pharmacies on drugs prescribed to individual patients [25].

Data on medication at hospital admission and hospital discharge were retrieved from the SWEDEHEART registry. Data regarding filled prescriptions for medications 6 months before hospital admission, and 1 and 6 months and 1–3 and 5 years after hospital admission, were retrieved from the Prescribed Drug Register.

Data on prescriptions for the following pharmaceuticals were included: acetylsalicylic acid (ATC-code B01AC06); P2Y12-inhibitors (B01AC04, B01AC22 and B01AC24); statins (C10AA and C10BA); beta blockers (C07); ACEs/ARBs including fixed combinations with thiazides (C09); Vitamin K antagonists (B01AA03); and novel oral anticoagulants (B01AE07, B01AF01, B01AF02 and B01AF03).

The study was approved by the Regional Ethical Review Board in Stockholm (diary number: 2012/60–31/2) and by the Swedish Ethical Review Authority (diary number: 2020–04252).

### Assessment of prescribing and medication adherence

All three constructs of adherence to medication, namely initiation, implementation and persistence, were evaluated [26]. In assessing adherence to medication only patients who received their first prescription for the above-mentioned drugs at hospital discharge were included, to minimize selection bias, as the prevalence of medications at admission differed significantly in the MINOCA and MI-CAD cohorts. Patients with ongoing use of a certain drug class and those prescribed a certain drug class within 6 months prior to MI were excluded from analyses on that particular drug class; however, these patients were eligible for inclusion and analysis regarding prescription of other drug classes.

**Fig 2:**
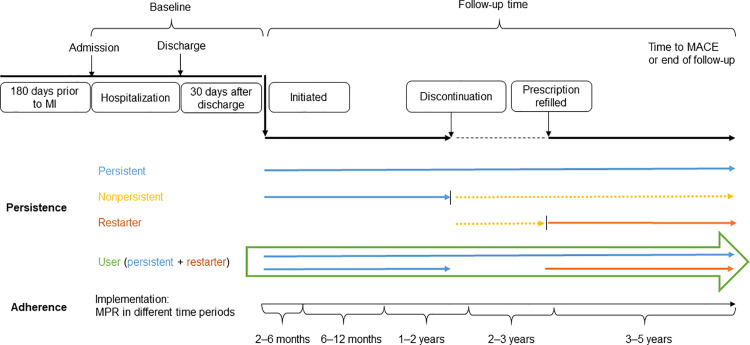
Study design. Time line demonstrating the times for initiation, implementation, and persistence of secondary preventive medications. Initiation; a filled prescription within 30 days after discharge. Persistence; the length of time between initiation and discontinuation of medical treatment (>45 days without refilled prescription). Non-persistent; patients discontinued treatment. Restarter; patients restarting treatment after being considered non-persistent. Users; the sum of persistent and restarting patients. Implementation: the extent to which a patient’s actual dosing regimen corresponded to the prescribed dosing regimen.

The time of follow-up was divided into six periods, 2–6 months, 6–12 months, 1–2 years, 2–3 years and 3–5 years (Fig 2).

### Initiation

Initiation was defined as the percentage of patients who had a drug prescription from a physician and dispensed the drug at a pharmacy within 30 days after discharge. Only patients who initiated medication were included in further analyses of implementation, discontinuation and persistence of that drug class.

### Implementation

Drug implementation, defined as the extent to which a patient’s actual dosing regimen corresponded to the prescribed dosing regimen, was estimated by determining the medication possession ratio (MPR) [26,27]. Briefly, for each time-period, the number of days a drug was available was divided by the number of days in that time-period. Stockpiling was included. The proportion of days with drug available was categorized as <50%, 50–80% and 80–100%, with an MPR ≥ 80% defined as high implementation [6,9,11,12,26,28,29].

### Persistence

Persistence in the present study was defined as the length of time between initiation and discontinuation of medical treatment. Patients were regarded as taking a drug as long as the prescription was refilled within the estimated time of the previous prescription, including drugs carried over from previous prescriptions. A grace period of 45 days was allowed, in which patients were considered continuously exposed to a drug if they refilled a prescription within 45 days after the estimated completion of previous prescriptions (Fig 2). The 45-day grace period were used to establish a reasonable balance between the need for monitoring short-term implementation and long-term persistence [27].

Patients were allowed to switch between drugs within the same drug class and still be considered persistent. If a patient failed to fill a new prescription within a given time, the date of non-persistence was defined as the calculated end of supply from the most recent prescription, including any stockpiling. On the first day of each interval, the proportion of persistent patients was calculated by dividing the number of persistent patients by the number of patients remaining in the cohort.

Patients who discontinued treatment were labeled non-persistent. Those who restarted treatment after being considered non-persistent were followed as a separate restarter group. The group users was defined as the sum of persistent and restarting patients. This provided an opportunity to capture patients restarting treatment after non-persistence and to calculate the actual proportion of patients receiving treatment at a certain time. The proportion of persistent patients at different time points was calculated by dividing the number of persistent patients by the number of patients remaining in the cohort at the first day of each interval,

Implementation was assessed only in patients who were persistent or users, to avoid confusing low implementation with non-persistence.

Patients who discontinued treatment and didn´t refill their prescription within 45 days were labeled non-persistent, whereas patients who continued to refill their prescription but took their medication less than 80% of the days were labeled persistent with low implementation.

### Statistics

Normally distributed continuous variables were presented as mean ± standard deviation (SD) and compared by Students’ t-tests, whereas non normally distributed continuous variables were presented as median and inter quartile range (IQR) and compared by Mann Whitney U-tests. Categorical variables were presented as frequencies and compared by Chi-square test. Multivariable logistic regression analyses were performed to investigate the association between MINOCA/MI-CAD status and the persistence of included medications at 12 months, adjusted for previously established cardiovascular risk factors like age, BMI, smoking, previous MI, hypertension, heart failure, diabetes, kidney failure, PVD, stroke, and COPD. The model for statins was also adjusted for non-HLD cholesterol. Logistic and linear regressions including implementation and persistence data, as well as different cardiovascular risk factors, were used as exploratory sensitivity analyses.

Statistical analyses were performed using SAS Software Version 9.4 (SAS Institute, Cary, NC, USA) and the Predictive Analytical SoftWare (PASW statistics 17.03) program (SPSS Inc, Chicago, IL, USA). All statistical tests were two-tailed, with p < 0.05 regarded as statistically significant.

## Results

A total of 9,138 patients diagnosed with MINOCA and 107,240 diagnosed with MI-CAD were followed-up for a mean 5.9 years. MINOCA patients were more often younger women with few risk factors for cardiovascular disease (Table 1).

**Table 1:** Baseline demographic and clinical characteristics of the study population.

	MINOCA	MI-CAD	p-value*
**Total, n**	9138	107240	
**Demographics**			
Female, n (%)	5774 (63.2)	30191 (28.2)	<0.001
Age, y, mean (±SD)	66 (11.6)	67 (11.4)	0.013
**Risk factors, n (%)**			
Smoking			<0.001
Never	4099 (44.9)	40742 (38.0)	
Previous	2986 (32.7)	35004 (32.7)	
Current	1672 (18.3)	27464 (25.6)	
Unknown	374 (4.1)	3936 (3.7)	
Diabetes	1144 (12.5)	20008 (18.7)	<0.001
Hypertension	1815 (19.9)	21397 (20.0)	0.836
BMI kg/m^2^, mean (±SD)	26.9 (9.3)	27.2 (5.2)	<0.001
**Medical history, n (%)**			
COPD	805 (8.8)	5162 (4.8)	<0.001
Kidney failure	101 (1.1)	1690 (1.6)	<0.001
Heart failure	301 (3.3)	2805 (2.6)	<0.001
Previous MI	137 (1.5)	4030 (3.8)	<0.001
Previous CABG	0	2845 (2.7)	<0.001
Previous PCI	0	2654 (2.5)	<0.001
PVD	169 (1.8)	3208 (3.0)	<0.001
Stroke	405 (4.4)	5678 (5.3)	<0.001
**Laboratory findings**			
Non-HDL mmol/L, mean (±SD)	3.6 (1.1)	3.9 (1.2)	<0.001
**ECG at presention, n (%)**			
ST-elevation	1234 (13.6)	44502 (41.7)	<0.001
Atrial fibrillation	728 (8.0)	6131 (5.7)	<0.001
**LVEF during hospital stay, n (%)**			<0.001
≥50%	5639 (74.3)	55556 (60.6)	
40-49%	1074 (14.1)	20306 (22.2)	
30-39%	566 (7.5)	11222 (12.2)	
<30%	247 (3.3)	3754 (4.1)	
Unknown	66 (0.9)	823 (0.9)	
**Medication prior admission, n (%)**			
Aspirin	1666 (18.2)	22662 (21.1)	<0.001
ACE-inhibitor or ARB	2679 (29.3)	29463 (27.5)	<0.001
Beta blocker	2184 (23.9)	25500 (23.8)	<0.001
DAPT	116 (6.6)	1795 (7.6)	0.134
Non-vitamin K anticoagulant	68 (0.7)	591 (0.6)	0.018
P2Y12-inhibitor	207 (2.3)	2805 (2.6)	0.043
Statin	1649 (18.1)	20752 (19.4)	<0.001
Warfarin	380 (4.2)	2958 (2.8)	<0.001
**Medication at discharge, n (%)**			
Aspirin	8053 (88.1)	103177 (96.2)	<0.001
ACEI/ARB	5914 (64.7)	86166 (80.3)	<0.001
Beta blocker	7335 (80.3)	97288 (90.7)	<0.001
DAPT	5950 (65.1)	92553 (86.3)	<0.001
Non-vitamin K anticoagulant	230 (2.5)	1818 (1.7)	<0.001
P2Y12-inhibitor	6264 (68.5)	95506 (89.1)	<0.001
Statin	7741 (84.7)	102383 (95.5)	<0.001
Warfarin	708 (7.8)	5534 (5.2)	<0.001
New prescriptions at discharge, n (%)**			
Aspirin	6474/7418 (87.3)	80303/83604 (96.1)	<0.001
ACEI/ARB	3320/6401 (51.9)	57099/76513 (74.6)	<0.001
Beta blocker	5209/6894 (75.6)	71593/80461 (89.0)	<0.001
Statin	6102/7444 (82.0)	81218/85538 (94.9)	<0.001
P2Y12-inhibitor	6053/8857 (68.3)	91792/103034 (89.1)	<0.001

* P-value: difference between MI-CAD and MINOCA.

** Prescriptions in patients without ongoing treatment or prescriptions 6 months prior myocardial infarction.

ACEI/ARB, ACE-inhibitor or angiotensin-receptor blocker; BMI, body mass index; CABG, coronary bypass grafting; COPD, chronic obstructive pulmonary disease; DAPT, dual antiplatelet therapy; LVEF, left ventricular ejection fraction; MI, myocardial infarction; PCI, percutaneous coronary intervention; PVD, peripheral vascular disease.

### Prescription and initiation

Patients with MINOCA were as expected less often prescribed and initiated on treatment with all assessed drug classes than patients with MI-CAD (S1 Table).

### Implementation

**Fig 3:**
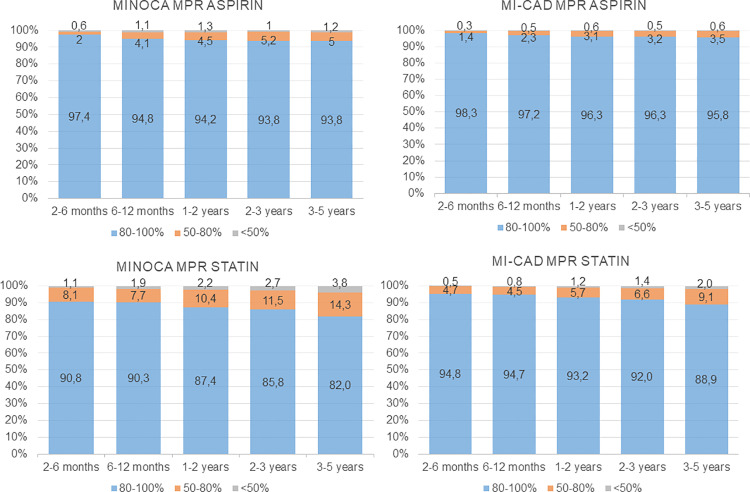
Implementation of aspirin and statins as secondary preventive treatment. Implementation, e.g., the extent to which a patient’s actual dosing regimen corresponded to the prescribed dosing regimen, in patients with MINOCA and MI-CAD. A medication possession ratio (MPR) ≥ 80% was defined as high implementation.

**Fig 4:**
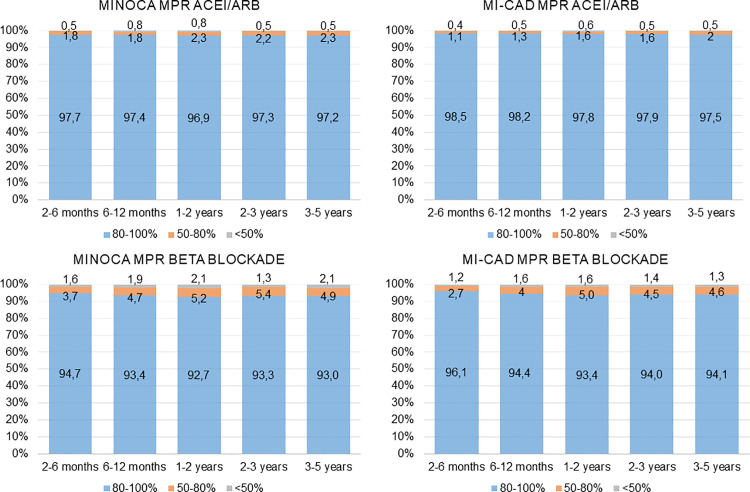
Implementation of ACEI/ARB and beta blockers as secondary preventive treatment. Implementation, e.g., the extent to which a patient’s actual dosing regimen corresponded to the prescribed dosing regimen, in patients with MINOCA and MI-CAD. A medication possession ratio (MPR) ≥ 80% was defined as high implementation.

Implementation, defined as the extent to which a patient’s actual dosing regimen corresponded to the prescribed dosing regimen, was highest at the beginning of follow-up and declined slowly over time. However, the proportions of patients with high implementation to treatment with aspirin, ACEI/ARBs, and beta blockers during all time periods were high in both the MINOCA and MI-CAD groups. The proportion of patients with high implementation to treatment with statins was lower in both the MINOCA and MI-CAD groups (Figs 3 and 4, S2 Table).

### Persistence

**Fig 5:**
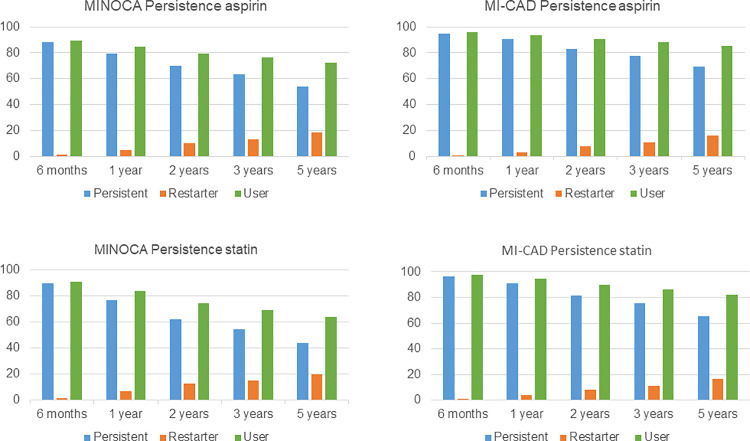
Persistence of treatment with aspirin and statins. Persistence; the length of time between initiation and discontinuation of medical treatment. Restarter; patients restarting treatment after being considered non-persistent. Users; the sum of persistent and restarting patients. On the first day of each interval, the proportion of persistent patients was calculated by dividing the number of persistent patients by the number of patients remaining in the cohort.

**Fig 6:**
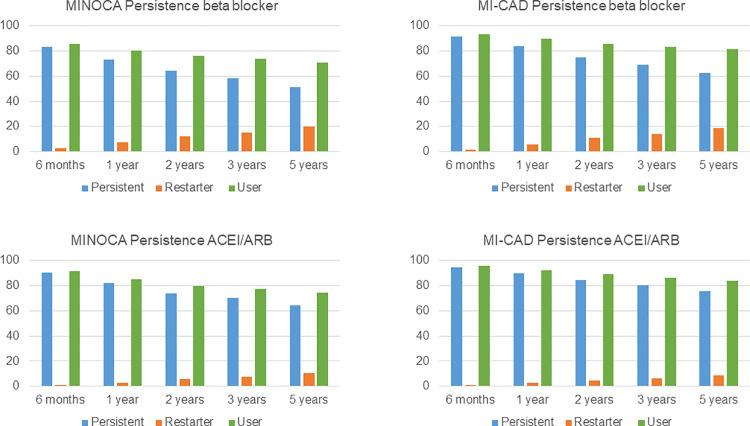
Persistence of treatment with ACEI/ARB and beta blockers. Persistence; the length of time between initiation and discontinuation of medical treatment. Restarter; patients restarting treatment after being considered non-persistent. Users; the sum of persistent and restarting patients. On the first day of each interval, the proportion of persistent patients was calculated by dividing the number of persistent patients by the number of patients remaining in the cohort.

Patients with MINOCA had lower persistence to all studied drug classes than patients with MI-CAD (Figs 5 and 6, S1 Table). The addition of restarting to persistent patients increased the rates of users of all classes of drugs, thus the difference between MINOCA and MI-CAD remained. Multivariable logistic regression analyses, after adjustment for relevant covariates, showed that persistence at 12 months remained significantly lower in the MINOCA than in the MI-CAD group (Table 2).

**Table 2:** Logistic regression models of factors associated with persistence of the investigated medications at 12 months.

		Number	Univariate regression	P-value	Multivariable regression	P-value
			OR (95% CI)		OR (95% CI)	
Aspirin	MI-CAD	68576	Ref.		Ref.	
	MINOCA	5556	0.365 (0.338-0.395)	<0.001	0.324 (0.299-0.358)	<0.001
Statins	MI-CAD	69730	Ref.		Ref.	
	MINOCA	5227	0.285 (0.263-0.309)	<0.001	0.327 (0.294-0.363)	<0.001
ACEI/ARBs	MI-CAD	2810	Ref.		Ref.	
	MINOCA	49015	0.478 (0.429-0.532)	<0.001	0.519 (0.461-0.584)	<0.001
Betablockers	MI-CAD	61734	Ref.		Ref.	
	MINOCA	4521	0.477 (0.441-0.515)	<0.001	0.467 (0.428-0.509)	<0.001

All multivariate analyses were adjusted for MINOCA/MI-CAD status, gender, age, BMI, smoking, previous MI, hypertension, heart failure, diabetes, kidney failure, PVD, stroke, and COPD. The model for statins was also adjusted for non-HLD cholesterol.

### Implementation and persistence in women

A subgroup analysis of women showed that rates of implementation of aspirin and statins were significantly higher in patients with MI-CAD than in those with MINOCA, whereas there were no difference in implementation rates of ACE/ARBs and beta blockers (Table 3). Persistence remained significantly higher in women with MI-CAD than in those with MINOCA (Table 4).

**Table 3:** Implementation of secondary preventive treatment in women with MINOCA and MI-CAD. A MPR ≥ 80% was defined as good adherence.

Aspirin	MINOCA	MI-CAD	p-value	Statin	MINOCA	MI-CAD	p-value
**MPR 2–6 months (n)**	3268	18774		**MPR 2–6 months (n)**	3099	18156	
80-100%	3197 (97.8%)	18467 (98.4%)	0.044	80-100%	2803 (90.4%)	18156 (94.0%)	<0.001
50-80%	55 (1.7%)	255 (1.4%)		50-80%	259 (8.4%)	1053 (5.4%)	
<50%	16 (0.5%)	52 (0.3%)		<50%	37 (1.2%)	113 (0.6%)	
**MPR 6–12 months (n)**	2874	17025		**MPR 6–12 months (n)**	2613	17277	
80-100%	2749 (95.7%)	16586 (97.4%)	<0.001	80-100%	2353 (90.0%)	16170 (93.6%)	<0.001
50-80%	98 (3.4%)	353 (2.1%)		50-80%	205 (7.8%)	935 (5.4%)	
<50%	27 (0.9%)	86 (0.5%)		<50%	55 (2.1%)	172 (1.0%)	
**MPR 1–2 years (n)**	2422	14579		**MPR 1–2 years (n)**	2022	14156	
80-100%	2297 (94.8%)	14154 (97.1%)	<0.001	80-100%	1746 (86.4%)	13016 (91.9%)	<0.001
50-80%	98 (4.0%)	368 (2.5%)		50-80%	228 (11.3%)	963 (6.8%)	
<50%	27 (1.1%)	57 (0.4%)		<50%	48 (2.4%)	177 (1.3%)	
**MPR 2–3 years (n)**	2069	12348		**MPR 2–3 years (n)**	1645	11668	
80-100%	1946 (94.1%)	11959 (96.8%)	<0.001	80-100%	1390 (84.5%)	10569 (90.6%)	<0.001
50-80%	106 (5.1%)	329 (2.7%)		50-80%	205 (12.5%)	903 (7.7%)	
<50%	17 (0.8%)	60 (0.5%)		<50%	50 (3.0%)	196 (1.7%)	
**MPR 3–5 years**	1468	8533		**MPR 3–5 years**	1077	7692	
80-100%	1377 (93.8%)	8268 (96.9%)	<0.001	80-100%	862 (80.0%)	6684 (86.9%)	<0.001
50-80%	75 (5.1%)	266 (2.6%)		50-80%	167 (15.5%)	838 (10.9%)	
<50%	16 (1.1%)	39 (0.5%)		<50%	48 (4.5%)	170 (2.2%)	
**ACEI/ARI**	**MINOCA**	**MI-CAD**	**p-value**	**Betablockade**	**MINOCA**	**MI-CAD**	**p-value**
**MPR 2–6 months (n)**	1684	12982		**MPR 2–6 months (n)**	2481	15824	
80-100%	1650 (98.0%)	12803 (98.6%)	0.011	80-100%	2353 (95.2%)	15253 (96.4%)	0.020
50-80%	29 (1.7%)	124 (1.0%)		50-80%	83 (3.3%)	396 (2.5%)	
<50%	5 (0.3%)	55 (0.4%)		<50%	35 (1.4%)	172 (1.1%)	
**MPR 6–12 months (n)**	1480	11661		**MPR 6–12 months (n)**	2190	14213	
80-100%	1442 (97.4%)	11453 (98.2%)	0.080	80-100%	2066 (94.3%	13525 (95.2%)	0.240
50-80%	27 (1.8%)	159 (1.4%)		50-80%	87 (4.0%)	493 (3.5%)	
<50%	11 (0.7%)	49 (0.4%)		<50%	37 (1.7%)	195 (1.4%)	
**MPR 1–2 years (n)**	1226	9912		**MPR 1–2 years (n)**	1848	12082	
80-100%	1197 (97.6%)	9720 (98.1%)	0.479	80-100%	1727 (93.5%)	11388 (94.3%)	0.371
50-80%	23 (1.9%)	142 (1.4%)		50-80%	91 (4.9%)	531 (4.4%)	
<50%	6 (0.5%)	50 (0.5%)		<50%	30 (1.6%)	163 (1.3%)	
**MPR 2–3 years (n)**	1052	8358		**MPR 2–3 years (n)**	1557	10260	
80-100%	1029 (97.8%)	8181 (97.9%)	0.361	80-100%	1473 (94.6%)	9752 (95.0%)	0.564
50-80%	19 (1.8%)	121 (1.4%)		50-80%	66 (4.2%)	380 (3.7%)	
<50%	4 (0.4%)	56 (0.7%)		<50%	18 (1.2%)	128 (1.2%)	
**MPR 3–5 years**	744	5674		**MPR 3–5 years**	1132	7151	
80-100%	723 (97.2%)	5559 (98.0%)	0.290	80-100%	1065 (94.1%)	6791 (95.0%)	0.326
50-80%	17 (2.3%)	86 (1.5%)		50-80%	49 (4.3%)	279 (3.9%)	
<50%	4 (0.5%)	29 (0.5%)		<50%	18 (1.6%)	81 (1.1%)	

*p-value compares persistent patients with MINOCA and persistent patient with MI-CAD. ACEI/ARB: ACE Inhibitor or angiotensin receptor blocker.

**Table 4:** Adherence to medication in women. Only patients with a de novo prescription of a drug class are included in analysis of that particular drug class. Patients with ongoing treatment or a prescription six months prior to myocardial infarction were excluded from analysis of that particular drug class.

	MINOCA				MI-CAD					
	Persistent	Restarters	Users	Cohort	Persistent	Restarters	Users	Cohort	p-value*	p-value**
**Statin**	4675				23887					
Prescribed, n (%)	3768 (80.6)				22167 (92.8)				<0.001	
Primary adherence	3587 (95.2)				21299 (96.1)				<0.001	
Persistens 2 months, n (%)	3523 (99.8)	0	3523 (99.8)	3529	20773 (99.7)	14 (0.1)	20787 (99.8)	20833	0.055	0.546
Persistens 6 months, n (%)	3052 (89.2)	51 (1.5)	3103 (90.7)	3422	19166 (95.6)	197 (1.0)	19363 (96.5)	20049	<0.001	<0.001
Persistens 1 year, n (%)	2455 (75.3)	224 (6.9)	2679 (82.2)	3259	16655 (88.4)	814 (4.3)	17469 (92.7)	18845	<0.001	<0.001
Persistens 2 year, n (%)	1727 (59.3)	374 (12.8)	2101 (72.1)	2913	12966 (77.6)	1472 ((8.8)	14438 (86.5)	16701	<0.001	<0.001
Persistens 3 year, n (%)	1325 (51.5)	385 (15.0)	1710 (66.4)	2574	10320 (71.1)	1661 (11.4)	11981 (82.5)	14522	<0.001	<0.001
Persistens 5 years, n (%)	793 (41.4)	376 (19.6)	1169 (61.0)	1917	6249 (60.1)	1763 (17.0)	8012 (77.0)	10401	<0.001	<0.001
**ASA**	4645				22862					
Prescribed, n (%)	4013 (86.4)				21757 (95.2)				<0.001	
Primary adherence	3817 (95.1)				20865 (95.9)				0.033	
Persistens 2 months, n (%)	3750 (100)	0	3750 (100)	3751	20376 (100)	0	20376 (100)	20388	0.094	0.491
Persistens 6 months, n (%)	3233 (89.2)	35 (1.0)	3268 (90.1)	3626	18617 (94.9)	155 (0.8)	18772 (95.7)	19616	<0.001	<0.001
Persistens 1 year, n (%)	2780 (80.8)	138 (4.0)	2918 (84.8)	3441	16610 (90.2)	580 (3.2)	17190 (93.4)	18412	<0.001	<0.001
Persistens 2 year, n (%)	2211 (71.8)	265 (8.6)	2476 (80.4)	3080	13688 (84.1)	1085 (6.7)	14773 (90.8)	16275	<0.001	<0.001
Persistens 3 year, n (%)	1771 (64.9)	344 (12.6)	2115 (77.5)	2730	11119 (78.6)	1378 (9.7)	12497 (88.4)	14142	<0.001	<0.001
Persistens 5 years, n (%)	1146 (55.6)	370 (17.9)	1516 (73.5)	2063	7224 (70.8)	1468 (14.4)	8692 (85.2)	10202	<0.001	<0.001
**Beta blocker**	4228				20849					
Prescribed, n (%)	3174 (75.1)				18490 (88.7)				<0.001	
Primary adherence	3030 (95.5)				17838 (96.5)				<0.001	
Persistens 2 months, n (%)	2962 (99.3)	0	2962 (99.3)	2982	17427 (99.8)	3 (0)	17430 (99.8)	17458	<0.001	<0.001
Persistens 6 months, n (%)	2419 (83.8)	66 (2.3)	2485 (86.0)	2888	15569 (92.5)	268 (1.6)	15837 (94.1)	16831	<0.001	<0.001
Persistens 1 year, n (%)	2056 (74.5)	181 (6.6)	2237 (81.1)	2758	13569 (85.7)	846 (5.3)	14415 (91.1)	15827	<0.001	<0.001
Persistens 2 year, n (%)	1638 (66.3)	271 (11.0)	1909 (77.3)	2470	10941 (77.9)	1418 (10.1)	12359 (88.0)	14044	<0.001	<0.001
Persistens 3 year, n (%)	1324 (60.7)	288 (13.2)	1612 (73.9)	2182	8920 (72.8)	1594 (13.0)	10514 (85.8)	12250	<0.001	<0.001
Persistens 5 years, n (%)	890 (53.5)	308 (18.5)	1198 (72.0)	1665	5853 (66.4)	1528 (17.3)	7381 (83.7)	8814	<0.001	<0.001
**ACEI/ARB**	3985				20711					
Prescribed, n (%)	2034 (51.0)				15085 (72.8)				<0.001	
Primary adherence	1935 (95.1)				14506 (96.2)				<0.001	
Persistens 2 months, n (%)	1909 (100)	0	1909 (100)	1909	14173 (88.0)	3 (0)	14176 (88.0)	16108	0.034	0.078
Persistens 6 months, n (%)	1673 (90.3)	11 (0.6)	1684 (90.9)	1853	12879 (94.3)	(0.9)	12999 (95.2)	13659	<0.001	<0.001
Persistens 1 year, n (%)	1450 (81.5)	42 (2.4)	1492 (83.8)	1780	11465 (89.1)	290 (2.3)	11755 (91.4)	12865	<0.001	<0.001
Persistens 2 year, n (%)	1172 (76.8)	81 (5.3)	1180 (77.3)	1526	9538 (83.7)	509 (4.5)	10047 (88.1)	11399	<0.001	<0.001
Persistens 3 year, n (%)	981 (70.1)	93 (6.6)	1074 (76.7)	1400	7880 (84.3)	592 (6.3)	7939 (84.9)	9352	<0.001	<0.001
Persistens 5 years, n (%)	664 (63.6)	111 (10.6)	775 (74.2)	1044	5243 (74.2)	562 (8.0)	5805 (82.2)	7064	<0.001	<0.001
**P2Y12-inhibitor**	5587				28882					
Prescribed, n (%)	3746 (67.0)				26003 (90.0)				<0.001	
Primary adherence	3535 (94.4)				25003 (96.2)				<0.001	
Persistens 2 months, n (%)	3478 (100)	0	3478 (100)	3479	24442 (100)	0	24442 (100)	24443	0.012	0.108
Persistens 6 months, n (%)	1313 (38.8)	26 (0.8)	1339 (39.6)	3385	18110 (77.2)	168 (0.7)	18278 (77.9)	23465	<0.001	<0.001
Persistens 1 year, n (%)	593 (18.4)	61 (1.9)	654 (20.3)	3219	12275 (55.8)	573 (2.6)	12848 (58.5)	21981	<0.001	<0.001

## Discussion

This nationwide registry-based study investigated and compared the initiation, implementation and persistence rates of secondary preventive medications in patients with MINOCA and MI-CAD. Patients with MINOCA were less frequently prescribed secondary preventive medications at discharge, showed a lower rate of filling their first prescriptions, and had lower implementation and persistence rates than patients with MI-CAD.

The proportion of patients with high implementation decreased slowly over time, although >90% of patients in both groups initiated on aspirin, beta blockers, and ACEI/ARBs maintained a MPR ≥ 80% during the entire follow-up period. The decreasing proportion of patients taking these medications over time is in agreement with several previous studies in patients with MI [6,9,10,12]. A recent study of statin implementation among patients with atherosclerotic cardiovascular disease showed that only 21.4% had high implementation during the first year, decreasing to 19.8% at 3 years [28]. The different results between our study and this study may be due in part to different compositions of study cohorts and methodological differences in assessing implementation. The present study only measured implementation in patients who were persistent or labeled as users both at the beginning and the end of a time period, to avoid mix up non-implementation and non-persistence, whereas previous studies did not. Furthermore, implementation in the present study was calculated using shorter time intervals at the start of follow-up because change of medications, side effects, and subsequent discontinuation may be more frequent at the beginning of treatment.

The present study found that the persistence of aspirin and statins in patients with MINOCA was in agreement with the results of previous studies assessing the persistence in MI patients at 12–18 months [5,7,8]. The rates of persistence of all medications throughout the entire follow-up period were higher in the present MI-CAD cohort than in previous studies [5,7,8]. The latter results are in agreement with a previous Swedish study investigating the long-term use of low-dose aspirin for both primary- and secondary prevention, with approximately 15% of those patients discontinuing long-term aspirin treatment after 3 years [30]. In contrast, the proportion of MINOCA patients in the present study who discontinued aspirin treatment was higher. However, the previous study found that patients who discontinued aspirin had a 37% higher rate of cardiovascular events after 3 years than those who were persistent [30]. The applicability of these findings to patients with MINOCA remains to be determined.

Several principal differences between patients with MINOCA and MI-CAD may affect the initiation, implementation, and persistence of secondary preventive medical treatment. First, the uncertainty of the diagnosis of MINOCA may affect both the attending physicians and patients’ willingness to prescribe medications and follow the prescription, respectively. The cause of MINOCA remains unclear in many patients [2,20,31,32]. Thus, patients with MINOCA are less likely to be prescribed secondary preventive medications, less often undergo structured follow-up, and less frequently achieve secondary preventive targets than patients with MI-CAD [33,34].

As recent guidelines recommend all patients with an initial working diagnosis of MINOCA follow a diagnostic algorithm, including a cardiac magnetic resonance (CMR) exam, to determine the underlying diagnosis [2,20] the previously experienced uncertainty should decrease with time. Henceforth, a CMR exam with an ischemic late gadolinium enhancement pattern that strengthens the indication for secondary preventive medical treatment, as it carries a more serious prognosis than a non-ischemic pattern, may improve both the prescription of secondary preventive medications and follow-up [35–38].

Second, the characteristics of patients with MINOCA differ from those with traditional MI. MINOCA patients tend to be younger, are more often women, and have fewer traditional risk factors for atherosclerotic heart disease [14,15,39]. Women with MI have been found to be less likely than men to receive evidence-based therapies and have lower referral rates for cardiac rehabilitation [5, 13, 40, 41]. Our subgroup analysis on women showed that the implementation of aspirin and statins were significantly higher in patients with MI-CAD than in those with MINOCA, whereas there was no difference in implementation rates of ACE/ARBs and beta blockers. Furthermore, persistence rates remained significantly higher in women with MI-CAD than in women with MINOCA, indicating that factors other than gender are important. Gender, however, may have a larger impact on the implementation and persistence of statins as perceived muscle symptoms associated with statin use are more common in women than in men [42,43].

None of the MINOCA patients in the present study had undergone a coronary intervention. MI patients treated without PCI are less frequently prescribed secondary preventive drugs than patients who undergo PCI (7). Prior cardiovascular treatment has also been associated with high long-term implementation of secondary preventive treatment [44]. In contrast, patients with asymptomatic disease may be less adherent [13,45].

Psychosocial factors may differ in patients with MINOCA and MI-CAD. Previous Swedish studies have indicated that pre-existing psychiatric disorders are more common in patients with MINOCA [46,47]. Moreover, patients with MINOCA were found to have lower rates in the dimensions of vitality and mental health at 3 months follow-up than patients with MI-CAD [46,47]. Other psychosocial factors, such as perceived social support and sense of coherence, have been associated with long-term adherence to secondary preventive measures in patients with MI [48]. Psychological belief and attitude are important in unintentional non-adherence, and beliefs about medication are important in intentional non-adherence [49].

A recent consensus document discussing adherence to secondary preventive therapy after cardiovascular diseases, recommended focus on all the five dimensions of adherence to therapy simultaneously; including the patient, the disease, the therapy, the healthcare provider and the healthcare system [13]. Thus, improving medical adherence requires both time and commitment. Novel interventions like digital health tools and follow-up programs that are both structured and individualized may contribute to an improvement of future secondary preventive medical treatment after both MINOCA and MI-CAD, but should preferably be studied in randomized trials.

### Strengths and limitations

This nationwide registry-based study included data from almost all patients hospitalized in Sweden for acute MI in 2006–2017, allowing analyses of complete and unselected patient cohorts. These findings reflect real-life practice as opposed to the setting of randomized controlled trials, thereby increasing the generalizability of the results. The use of registry reduces potential selection bias associated with studies of patients at selected hospitals or enrolled in health care insurance systems. Furthermore, restricting the assessment of implementation and persistence only to patients who had a de novo prescription for each indicated class of drugs reduced the influence of on-going prescriptions on long-term persistence.

However, this registry-based analysis had several limitations. The analysis relied on ICD-codes and the possibility of coding errors cannot be ruled out. Diagnostic criteria for MINOCA were not proposed until 2017 [19], making it impossible to determine how many patients, who today would meet the criteria for MINOCA, were diagnosed with a non-MI related condition. Furthermore, CMR imaging was not used to the same extent during the study period as today and it is possible some of the patients labelled as MINOCA in this study in fact had an undiagnosed Takotsubo cardiomyopathy or myocarditis [35].

The secondary preventive medication after MI recommended in guidelines have been similar during the study period, e.g., class 1 recommendations for aspirin, beta blockers, ACEI/ARB and statins [50], whereas recommendations specific for MINOCA weren’t published until after the study period [2,20]. The physician’s prescription pattern of secondary preventive medications to patients with MINOCA may therefore vary over time. However, to minimize the impact of the prescription patterns the present study only included patients prescribed and initiating medication in further analyses of implementation and persistence.

The Swedish Prescribed Drug Register records complete data of prescribed drugs dispensed to individuals. However, it do not contain information how many patients who were prescribed drugs did not collect them or the number of those collecting the drugs who did not take them.

In addition, the lack of information on patient socioeconomic status and previous psychiatric illnesses may have resulted in residual confounding, as factors such as low economic status, low education status and psychiatric disease are previously described barriers to adherence to treatment [13].

The differences between this study and previous studies in the methods used to measure implementation and persistence make it difficult to compare results. Compared with many previous studies, the present study applied a stricter initial definition, measuring implementation and persistence only in patients with primary adherence to treatment, but a less rigid follow-up approach including patients who restarted treatment in the user group. Both of these factors may have resulted in higher levels of persistence at later time points than observed with other approaches, but may better reflect real world conditions.

## Conclusions

This nationwide study demonstrated that the rates of initiation, implementation, and persistence of secondary preventive medications were high in both MINOCA and MI-CAD patients during the first 5 years after MI. These rates, however, were lower in patients with MINOCA, a difference that may be partially due to uncertainties regarding the diagnosis of MINOCA, differences in patient characteristics, and psychosocial factors. Suboptimal medical adherence in patients with MINOCA may adversely affect prognosis as previously demonstrated in MI-CAD patients.

## Supporting information

S1 Table Adherence to medication. On the first day of each interval, the proportion of persistent patients was calculated by dividing the number of persistent patients by the number of patients remaining in the cohort. Only patients with a de novo prescription of a drug class were included in analysis of that drug class. Patients with ongoing treatment or a prescription 6 months prior to myocardial infarction were excluded from analysis of that drug class.
(DOCX)

S2 Table Implementation of secondary preventive treatment with aspirin, statins, ACEI/ARBs, and beta blockers in patients with MINOCA and MI-CAD. Medication possession ratio (MPR) ≥80% was defined as high adherence.
(DOCX)

## Abbreviations

ACEI: angiotensin-converting enzyme inhibitor
ARB: angiotensin-receptor blocker
MI-CAD: myocardial infarction with obstructive coronary arteries
MINOCA: myocardial infarction with non-obstructive coronary arteries.
