# Supplementary material for: Adherence to secondary preventive treatment following myocardial infarction with and without obstructive coronary artery disease
Source: PLoS One. 2025 May 23;20(5):e0324072. doi: 10.1371/journal.pone.0324072 (PMC12101741; doi:10.1371/journal.pone.0324072)
Supplement: S1 Table — On the first day of each interval, the proportion of persistent patients was calculated by dividing the number of persistent patients by the number of patients remaining in the cohort. Only patients with a de novo prescription of a drug class were included in analysis of that drug class. Patients with ongoing treatment or a prescription 6 months prior to myocardial infarction were excluded from analysis of that drug class. (DOCX) [file pone.0324072.s001.docx]

**Supplemental table 1. Adherence to medication.** Only patients with a de novo prescription of a drug class were included in analysis of that drug class. Patients with ongoing treatment or a prescription 6 months prior to myocardial infarction were excluded from analysis of that drug class.

|  | **MINOCA** |  |  |  | **MI-CAD** |  |  |  |
| --- | --- | --- | --- | --- | --- | --- | --- | --- |
|  | **Cohort** | **Users*** | **Persistent †** | **Restarters** | **Cohort** | **Users *** | **Persistent †** | **Restarters** |
|  |  |  |  |  |  |  |  |  |
| **Statin, n (%)** |  |  |  |  |  |  |  |  |
| Discharged alive | 7444 |  |  |  | 85538 |  |  |  |
| Prescribed | 6102 |  |  |  | 81218 |  |  |  |
| Initiated |  | 5799 |  |  |  | 78244 |  |  |
| At 6 months | 5518 | 5022 (91.0%) | 4946 (89.6%) | 76 (1.4%) | 73923 | 71996 (97.4%) | 71394 (96.6%) | 602 (0.8%) |
| At 1 year | 5257 | 4403 (83.8%) | 4041 (76.9%) | 362 (6.9%) | 69730 | 66079 (94.8%) | 63399 (90.9%) | 2680 (3.8%) |
| At 2 year | 4699 | 3508 (74.7%) | 2907 (61.9%) | 601 (12.8%) | 61884 | 55499 (89.7%) | 50534 (81.7%) | 4965 (8.0%) |
| At 3 year | 4177 | 2900 (69.4%) | 2271 (54.4%) | 629 (15.1%) | 54222 | 46881 (86.5%) | 40981 (75.6%) | 5900 (10.9%) |
| At 5 years | 3115 | 1993 (64.0%) | 1371 (44.0%) | 622 (20.0%) | 39377 | 32383 (82.2%) | 25756 (65.4%) | 6627 (16.8%) |
| **Aspirin, n (%)** |  |  |  |  |  |  |  |  |
| Discharged alive | 7418 |  |  |  | 83604 |  |  |  |
| Prescribed | 6474 |  |  |  | 80303 |  |  |  |
| Initiated |  | 6144 |  |  |  | 77109 |  |  |
| At 6 months | 5838 | 5207 (89.2%) | 5140 (88.0%) | 67 (1.1%) | 72793 | 69715 (95.8%) | 69113 (94.9%) | 602 0.8%) |
| At 1 year | 5556 | 4687 (84.4%) | 4403 (79.2%) | 284 (5.1%) | 68576 | 64225 (93.7%) | 61975 (90.4%) | 2250 (3.3%) |
| At 2 year | 4960 | 3949 (79.6%) | 3459 (69.7%) | 490 (9.9%) | 60852 | 55166 (90.7%) | 50594 (83.1%) | 4572 (7.5%) |
| At 3 year | 4394 | 3361 (76.5%) | 2775 (63.2%) | 586 (13.3%) | 53317 | 47139 (88.4%) | 41376 (77.6%) | 5763 (10.8%) |
| At 5 years | 3301 | 2387 (72.3%) | 1780 (53.9%) | 607 (18.4%) | 38872 | 33186 (85.4%) | 26990 (69.4%) | 6196(15.9%) |
| **Beta blocker, n (%)** |  |  |  |  |  |  |  |  |
| Discharged alive | 6894 |  |  |  | 80461 |  |  |  |
| Prescribed | 5209 |  |  |  | 71593 |  |  |  |
| Initiated |  | 4968 |  |  |  | 69089 |  |  |
| At 6 months | 4734 | 4064 (85.8%) | 3943 (83.3%) | 121 (2.6%) | 65408 | 61004 (93.3%) | 59799 (91.4%) | 1205 (1.8%) |
| At 1 year | 4521 | 3633 (80.4%) | 3309 (73.2%) | 324 (7.2%) | 61734 | 55289 (89.6%) | 51593 (83.6%) | 3696 (6.0%) |
| At 2 year | 4051 | 3090 (76.3%) | 2597 (64.1%) | 493 (12.2%) | 54939 | 47108 (85.8%) | 41052 (74.7%) | 6056 (11.0%) |
| At 3 year | 3605 | 2650 (73.5%) | 2102 (58.3%) | 548 (15.2%) | 48253 | 40302 (83.5%) | 33460 (69.3%) | 6842 (14.2%) |
| At 5 years | 2727 | 1935 (71.0%) | 1391 (51.0%) | 544 (20.0%) | 35244 | 28684 (81.4%) | 21998 (62.4%) | 6686 (19.0%) |
| **ACEI/ARB, n (%)** |  |  |  |  |  |  |  |  |
| Discharged alive | 6401 |  |  |  | 76519 |  |  |  |
| Prescribed | 3320 |  |  |  | 57099 |  |  |  |
| Initiated |  | 3159 |  |  |  | 54952 |  |  |
| At 6 months | 3007 | 2745 (91.3%) | 2716 (90.3%) | 29 (1.0%) | 51946 | 49616 (95.5%) | 49197 (94.7%) | 419 (0.8%) |
| Ats 1 year | 2870 | 2440 (85.0%) | 2354 (82.0%) | 86 (3.0%) | 49015 | 45206 (92.2%) | 43999 (89.8%) | 1207 (2.5%) |
| At 2 year | 2569 | 2044 (79.6%) | 1895 (73.8%) | 149 (5.8%) | 43582 | 38757 (88.9%) | 36698 (84.2%) | 2059 (4.7%) |
| At 3 year | 2254 | 1745 (77.4%) | 1578 (70.0%) | 167 (7.4%) | 38162 | 33020 (86.5%) | 30659 (80.3%) | 2361 (6.2%) |
| At 5 years | 1665 | 1240 (74.5%) | 1067 (64.1%) | 173 (10.4%) | 27615 | 23136 (83.8%) | 20800 (75.3%) | 2336 (8.5%) |
| **P2Y12-inhibitor, n (%)** |  |  |  |  |  |  |  |  |
| Discharged alive | 8857 |  |  |  | 103034 |  |  |  |
| Prescribed | 6053 |  |  |  | 91792 |  |  |  |
| Initiated |  | 5694 |  |  |  | 88275 |  |  |
| At 6 months | 5434 | 2235 (41.1%) | 2185 (40.2%) | 50 (0.9%) | 83128 | 65481 (78.8%) | 64745 (77.9%) | 736 (0.9%) |
| At 1 year | 5173 | 1146 (22.2%) | 1031 (19.9%) | 115 (22.2%) | 78139 | 46765 (59.8%) | 44499 (56.9%) | 2266 (29.0%) |
| All results presented as n (%). | |  |  |  |  |  |  |  |
| * the p-value for comparisons of user patient with MINOCA and MI-CAD are <0.001 for all drug groups at all time points. | | | | | | |  |  |
| † the p-value for comparisons of persistent patient with MINOCA and MI-CAD are <0.001 for all drug groups at all time points. | | | | | | |  |  |
| ACEI/ARB: ACE inhibitor or angiotensin receptor blocker. | | | |  |  |  |  |  |
