# Supplementary material for: Adherence to secondary preventive treatment following myocardial infarction with and without obstructive coronary artery disease
Source: PLoS One. 2025 May 23;20(5):e0324072. doi: 10.1371/journal.pone.0324072 (PMC12101741; doi:10.1371/journal.pone.0324072)
Supplement: S2 Table — Medication possession ratio (MPR) ≥80% was defined as high adherence. (DOCX) [file pone.0324072.s002.docx]

**Supplemental table 2.** Implementation of secondary preventive treatment with aspirin, statins, ACEI/ARBs, and beta blockers in patients with MINOCA and MI-CAD. Medication possession ratio (MPR) ≥80% was defined as high adherence.

| **Aspirin** | **MINOCA** | **MI-CAD** | **p-value** | **Statin** | **MINOCA** | **MI-CAD** | **p-value** |
| --- | --- | --- | --- | --- | --- | --- | --- |
| **MPR 2–6 months (n)** | 5209 | 69718 |  | **MPR 2–6 months (n)** | 5014 | 71892 |  |
| 80 -100% | 5074 (97.4%) | 68503 (98.3%) | <0.001 | 80 -100% | 4553 (90.8%) | 68146 (94.8%) | <0.001 |
| 50 -80% | 106 (2.0%) | 973 (1.4%) |  | 50 -80% | 406 (8.1%) | 3365 (4.7%) |  |
| **MPR 6–12 months (n)** | 4590 | 63517 |  | **MPR 6–12 months (n)** | 4288 | 65347 |  |
| 80 -100% | 4353 (94.8%) | 61725 (97.2%) | <0.001 | 80 -100% | 3874 (90.3%) | 61872 (94.7%) | <0.001 |
| 50 -80% | 188 (4.1%) | 1478 (2.3%) |  | 50 -80% | 332 (7.7%) | 2960 (4.5%) |  |
| **MPR 1–2 years (n)** | 3854 | 54381 |  | **MPR 1–2 years (n)** | 3372 | 54647 |  |
| 80 -100% | 3630 (94.2%) | 52390 (96.3%) | <0.001 | 80 -100% | 2947 (87.4%) | 50905 (93.2%) | <0.001 |
| 50 -80% | 172 (4.5%) | 1673 (3.1%) |  | 50 -80% | 350 (10.4%) | 3102 (5.7%) |  |
| **MPR 2–3 years (n)** | 3274 | 46448 |  | **MPR 2–3 years (n)** | 2789 | 45803 |  |
| 80 -100% | 3072 (93.8%) | 44724 (96.3%) | <0.001 | 80 -100% | 2394 (85.8%) | 42130 (92.0%) | <0.001 |
| 50 -80% | 169 (5.2%) | 1482 (3.2%) |  | 50 -80% | 320 (11.5%) | 3014 (6.6%) |  |
| **MPR 3–5 years (n)** | 2307 | 32457 |  | **MPR 3–5 years** | 1838 | 31131 |  |
| 80 -100% | 2165 (93.8%) | 31110 (95.8%) | <0.001 | 80 -100% | 1507 (82.0%) | 27667 (88.9%) | <0.001 |
| 50 -80% | 115 (5.0%) | 1146 (3.5%) |  | 50 -80% | 262 (14.3%) | 2847 (9.1%) |  |
| **ACEI/ARB** | **MINOCA** | **MI-CAD** | **p-value** | **Betablockers** | **MINOCA** | **MI-CAD** | **p-value** |
| **MPR 2–6 months (n)** | 2742 | 49562 |  | **MPR 2–6 months (n)** | 4056 | 60959 |  |
| 80 -100% | 2680 (97.7%) | 48836 (98.5%) | 0.002 | 80 -100% | 3841 (94.7%) | 58543 (96.0%) | <0.001 |
| 50 -80% | 48 (1.8%) | 525 (1.1%) |  | 50 -80% | 151 (3.7%) | 1666 (2.7%) |  |
| **MPR 6–12 months (n)** | 2408 | 44805 |  | **MPR 6–12 months (n)** | 3544 | 54440 |  |
| 80 -100% | 2345 (97.4%) | 43984 (98.2%) | 0.017 | 80 -100% | 3310 (93.4%) | 51382 (94.4%) | 0.045 |
| 50 -80% | 44 (1.8%9 | 602 (1.3%) |  | 50 -80% | 166 (4.7%) | 2204 (4.0%) |  |
| **MPR 1–2 years (n)** | 1996 | 38200 |  | **MPR 1–2 years (n)** | 2967 | 45907 |  |
| 80 -100% | 1933 (96.9%) | 37374 (97.8%) | 0.020 | 80 -100% | 2751 (92.7%) | 42888 (93.4%) | 0.108 |
| 50 -80% | 46 (2.3%) | 612 (1.6%) |  | 50 -80% | 154 (5.2%) | 2283 (5.0%) |  |
| **MPR 2–3 years (n)** | 1711 | 32547 |  | **MPR 2–3 years (n)** | 2544 | 39249 |  |
| 80 -100% | 1664 (97.3%) | 31850 (97.9%) | 0.163 | 80 -100% | 2373 (93.3%) | 36913 (94.0%) | 0.110 |
| 50 -80% | 38 (2.2%) | 527 (1.6%) |  | 50 -80% | 137 (5.4%) | 1768 (4.5%) |  |
| **MPR 3–5 years (n)** | 1196 | 22624 |  | **MPR 3–5 years (n)** | 1832 | 27632 |  |
| 80 -100% | 1162 (97.2%) | 22056 (97.5%) | 0.680 | 80 -100% | 1704 (93.0%) | 25997 (94.1%) | 0.022 |
| 50 -80% | 28 (2.3%) | 448 (2.0%) |  | 50 -80% | 90 (4.9%) | 1269 (4.6%) |  |
| All results presented as n (%).  *p-values for comparisons of persistent patients with MINOCA and MI-CAD. ACEI/ARB:  ACE inhibitor or angiotensin receptor blocker. | | | | | | |  |
